# Tissue-specific DNA methylation profiles in newborns

**DOI:** 10.1186/1868-7083-5-8

**Published:** 2013-05-31

**Authors:** Emilie Herzog, Jubby Galvez, Anton Roks, Lisette Stolk, Michael Verbiest, Paul Eilers, Jan Cornelissen, Eric Steegers, Régine Steegers-Theunissen

**Affiliations:** 1 Department of Obstetrics and Gynaecology, Erasmus MC, University Medical Centre Rotterdam, dr. Molewaterplein 50, Rotterdam, GE 3015, the Netherlands; 2 Department of Internal Medicine, Section of Vascular Medicine and Pharmacology, Erasmus MC, University Medical Centre Rotterdam, dr. Molewaterplein 50, Rotterdam, GE 3015, the Netherlands; 3 Department of Internal Medicine, Erasmus MC, University Medical Centre Rotterdam, dr. Molewaterplein 50, Rotterdam, GE 3015, the Netherlands; 4 Department of Biostatistics, Erasmus MC, University Medical Centre Rotterdam, dr. Molewaterplein 50, Rotterdam, GE 3015, the Netherlands; 5 Department of Haematology, Erasmus MC, University Medical Centre Rotterdam, dr. Molewaterplein 50, Rotterdam, GE 3015, the Netherlands; 6 Department of Clinical Genetics, Erasmus MC, University Medical Centre Rotterdam, dr. Molewaterplein 50, Rotterdam, GE 3015, the Netherlands

**Keywords:** Epigenetics, Umbilical cord blood, Wharton jelly, Placenta, *IGF2*/*H19*

## Abstract

**Background:**

Epidemiological studies demonstrate that foetal growth restriction and low birth weight affect long-term health. Derangements in tissue-specific epigenetic programming of foetal and placental tissues are a suggested underlying mechanism of which DNA methylation is best understood. DNA methylation has been mostly investigated in DNA from white blood cells. To improve baseline understanding of tissue-specific DNA methylation, we examined variation in DNA methylation profiles of the imprinted foetal growth genes *IGF2* and *H19* in three different tissues from the same newborn obtained at the same time.

**Findings:**

We obtained DNA from umbilical cord blood mononuclear cells (MNC; CD34^+^ and CD34^–^, *n* = 6), foetal side of the placenta (*n* = 5) and umbilical cord Wharton jelly (*n* = 5). DNA methylation of the *IGF2* differentially methylated region (*DMR*) and *H19 DMR* was measured using quantitative mass spectrometry. Analysis of variance testing showed no statistical difference between total mean methylation of CD34^+^ and CD34^–^ MNC. Further comparisons were made with the pooled total MNC fraction. Mean *IGF2 DMR* methylation of Wharton jelly was 1.3 times higher (*P* = 0.001) than mean methylation of the pooled MNC. Placental mean methylation was 0.8 times lower (*P* <0.001) and Wharton jelly 0.9 times lower (*P* <0.001) than the pooled MNC of *H19 DMR.*

**Conclusion:**

The total MNC fraction is a rather homogeneous cell population for methylation studies of imprinted genes in umbilical cord blood white blood cells, but may not always reflect the methylation levels of *IGF2* and *H19* in other organs.

## Findings

### Background

The prenatal period is critical for adverse pregnancy outcome and chronic diseases in adulthood [1]. Epigenetic programming of foetal and placental tissues is a suggested underlying mechanism, of which DNA methylation is best understood [2,3].

DNA methylation profiles are tissue specific in somatic and germline tissues [2,4,5]. This is important in the tissue-specific regulation of cellular differentiation and lineage maintenance [6-8]. However, human methylation profiles are mostly performed in DNA from an easily accessible, heterogeneous white blood cell population. DNA methylation studies often select imprinted loci as candidate genes, because DNA methylation levels at these loci were assumed to be comparable in different tissues. Recent literature, however, has questioned this assumption [2,9,10]. The imprinted *IGF2–H19* gene complex, involved in placental, embryonic and foetal growth and development, has been described extensively in this context. Both genes are located near each other and are reciprocally imprinted [11]. Studies have demonstrated in mice that placental *Igf2* knockout results in foetal growth restriction, whereas *H19* silencing leads to foetal overgrowth [12,13]. In human, the phenotype related to the silencing of *IGF2* is Silver Russell syndrome and *H19* silencing is related to Beckwith Wiedemann syndrome [9].

From this background, we aimed to improve the baseline understanding of tissue-specific variation in DNA methylation profiles of the imprinted genes *IGF2* and *H19*, and therefore examined umbilical cord blood mononuclear cells (MNC), placental tissue and Wharton jelly derived from the umbilical cord. The rationale for selecting these tissues is that they are easily accessible, MNC consist of a rather homogeneous population of white blood cells, and placental and umbilical cord tissues are involved in foetal programming and development. Moreover, morphological abnormalities in these tissues are related to pregnancy complications, in which epigenetic derangements might be involved [14-17]. To examine a possible methylation difference between MNC subpopulations, CD34^+^ and CD34^–^ fractions were also analysed separately.

## Methods

### Maternal, pregnancy and child characteristics

In this study we analysed samples of six pregnancies. Median maternal age was 30.5 years (range: 23.8 to 37.3) and median parity was 0.5 (0 to 2). All pregnancies were uncomplicated, except one gestational hypertension (peak blood pressure: 140/90 mmHg). Deliveries were at term and spontaneously, median birth weight was 3,303 g (2,795 to 3,975). Two out of six newborns were male. Samples were collected after written informed consent was obtained before delivery at the Erasmus MC, University Medical Centre Rotterdam, the Netherlands. Ethical approval was given by the Erasmus MC, University Medical Centre Research Ethics Board (MEC-2004-227).

### Sample collection

Immediately after delivery of the newborn with the placenta still *in situ*, umbilical cord blood (*n* = 6) was collected in cord blood collection bags containing 21 ml anticoagulant citrate phosphate dextrose solution. The placenta (*n* = 5) and umbilical cord (*n* = 5) were collected within 10 minutes after delivery of the placenta. Samples of 0.5 cm^3^ were taken from the foetal side of the placental villi at four different sites in a 3 cm radius around the umbilical cord insertion, after carefully removing the membranes and 2 mm of the top placental layer. Wharton jelly from the umbilical cord was isolated in pieces of 0.5 cm^2^ avoiding the umbilical cord vessels. Tissues were frozen immediately in liquid nitrogen and stored at −80°C until DNA extraction. All samples were collected by two researchers.

### Blood cell separation

Umbilical cord blood was processed within 48 hours after collection. Using Ficoll gradient centrifugation, the MNC fraction was obtained and washed. CD34^+^ MNC were isolated from this pool by magnetic-activated cell separation using a Direct CD34 Progenitor Cell Isolation Kit (130-046-702; Miltenyi Biotec, Bergisch Gladbach, Germany) according to the manufacturer’s protocols. The remaining cells were collected and further analysed as CD34^–^ MNC.

### DNA extraction

Placental and Wharton jelly tissues were ground on liquid nitrogen and lysed overnight at 55°C using cell lysis buffer. Subsequently, genomic DNA was extracted from all tissues using the Gentra Puregene Tissue Kit (Qiagen, Hilden, Germany), following the manufacturer’s instructions.

### DNA methylation measurement

The amplicons for *IGF2* and *H19* have been described previously [18]. The amplicon for *IGF2* is located in the *IGF2* differentially methylated region (*DMR*), upstream of exon 1 of *IGF2*. For *H19*, the amplicon partly overlaps a CpG island, which is part of the *H19 DMR*, upstream of exon 1 of *H19*. Table 1 shows the location, length and primers of the amplicons. Firstly, the amplicons were tested on a standard curve constructed from DNA with low and high methylation (EpigenDx, Worcester, MA, USA) at stages of 10% methylation difference. Only amplicons with a good distribution of the methylation percentages were used for measurements of the samples.

**Table 1:** Characteristics of primers per gene

	**Forward primer**	**Reverse primer**	**Base pair length**	**Position**	**CpG sites ( *n *)**
*IGF2 DMR*	aggaagagagTGGATAGGAGATTGAGGAGAAA	cagtaatacgactcactatagggagaaggctAAACCCCAACAAAAACCACT	338	Chr. 11: 2,169,458 to 2,169,796	7
*H19 DMR*	aggaagagagGGGTTTGGGAGAGTTTGTGAGGT	cagtaatacg actcactata gggagaaggctATACCTACTACTCCCTACCTACCAAC	413	Chr. 11: 2,019,371 to 2,019,784	20

NCBI build: 37. Tags in lowercase. *Chr* chromosome, *DMR* differentially methylated region.

Isolated genomic DNA (500 ng) was treated with sodium bisulphite for 16 hours using the EZ-96 DNA methylation kit (Shallow; Zymo Research, Irvine, CA, USA). This was followed by PCR amplification, reverse transcription, fragmentation and analysis on a mass spectrometer (Sequenom, Inc., San Diego, CA, USA). This generated mass signal patterns that were translated into quantitative DNA methylation levels per CpG site by Mass ARRAY EpiTYPER Analyzer software (v1.0, build1.0.6.88; Sequenom, Inc.) [19]. Fragments containing one or more CpG sites were called CpG units. Measurements were performed in triplicate on DNA from the same bisulphite-treatment batch on different PCR plates. On every bisulphite plate, standard DNA with low, 25%, 50%, 75% and high methylation was included.

### Data cleaning

During quality control, CpG units with a very low mass or very high mass or CpG units with overlapping RNA fragments were excluded from further analysis. Two out of three of the replicate measurements per CpG unit had to be successful, and the standard deviation of the duplicates or triplicates had to be ≤0.10 to be included in the statistical analysis. CpG units with interference of SNPs were also excluded (dbSNP134). After quality control, 3 CpG units for *IGF2 DMR* and 9 CpG units for *H19 DMR* remained for further analysis.

### Statistical analysis

Possible batch effects were ruled out by comparing means of the standards per bisulphite plate and PCR plate with analysis of variance testing. To analyse total methylation per gene and per individual CpG unit between tissues, analysis of variance testing was used, followed by pairwise comparisons. We adjusted the total methylation per gene for the number of CpG units.

We checked and confirmed the normal distribution by visual inspection of the residuals. Several individual CpG sites showed significant differences in variance of DNA methylation. We excluded one patient and 4 CpGs from further testing for the *H19 DMR* to deal with this variation. Analysis of variance was finally performed on 3 CpG units of *IGF2 DMR* and 5 CpG units of *H19 DMR.*

Firstly, we analysed CD34^+^ and CD34^–^ MNC separately, followed by a weighted pooled total MNC fraction after these two fractions appeared not statistically differently methylated. The original CD34^+^ and CD34^–^ data were pooled in a 1:100 distribution, comparable with the biological appearance of CD34^+^ cells in an umbilical cord blood MNC fraction. Bonferroni correction was applied to correct for multiple comparisons. All tests were performed using means of the data in triplicate. Statistical analysis was performed in SPSS version 20.0 (SPSS, Inc., Chicago, IL, USA).

## Results

The mean methylation of CpG sites of the *IGF2 DMR* and the *H19 DMR* are depicted in Table 2. *IGF2 DMR* and *H19 DMR* methylation of CD34^+^ and CD34^–^ MNC were not statistically different, neither the total mean methylation per amplicon nor the individual CpG units. Further comparisons were therefore made with the weighted pooled total MNC fraction as a reference group. The mean *IGF2 DMR* methylation of Wharton jelly (*P* = 0.001) was statistically significantly higher than the mean methylation of MNC. This was similar in two out of the three individual *IGF2 DMR* CpG units.

**Table 2:** Absolute methylation levels of the different tissues per gene and per CpG site

	**CD34**^**+ **^**MNC**	**CD34**^**– **^**MNC**	**Pooled MNC**	**Placental tissue**	**Wharton jelly**
***IGF2 DMR *(total)**	**0.55 (0.14)**	**0.50 (0.13)**	**0.50 (0.13)**	**0.54 (0.16)**	0.65 (0.13)†*
*IGF2 DMR* CpG 3	0.59 (0.05)	0.52 (0.02)	0.52 (0.02)	0.59 (0.07)	0.66 (0.07)**†**
*IGF2 DMR* CpG 4	0.64 (0.18)	0.59 (0.18)	0.59 (0.18)	0.58 (0.25)	0.77 (0.11)
*IGF2 DMR* CpG 6.7	0.43 (0.06)	0.39 (0.03)	0.39 (0.03)	0.44 (0.03)	0.52 (0.06)**†**
***H19 DMR *(total)**	**0.30 (0.02)**	**0.31 (0.02)**	**0.31 (0.02)**	**0.25 (0.02)†**	0.28 (0.03)†*
*H19 DMR* CpG 2	0.28 (0.01)	0.29 (0.01)	0.29 (0.01)	0.26 (0.01)	0.27 (0.04)
*H19 DMR* CpG 9.10	0.31 (0.01)	0.31 (0.02)	0.31 (0.02)	0.26 (0.03)	0.30 (0.04)
*H19 DMR* CpG 12	0.28 (0.02)	0.29 (0.01)	0.29 (0.01)	0.23 (0.02)**†**	0.26 (0.02)**†**
*H19 DMR* CpG 13	0.30 (0.01)	0.31 (0.01)	0.31 (0.01)	0.24 (0.01)**†**	0.28 (0.03)
*H19 DMR* CpG 17	0.34 (0.02)	0.35 (0.01)	0.35 (0.01)	0.25 (0.03)**†**	0.31 (0.03)*

Data presented as mean (standard deviation). Analysis of variance testing between the different tissue groups. Bonferroni correction was applied to all *P* values to adjust for multiple comparisons. *DMR* differentially methylated region; MNC mononuclear cells.

**†***P* <0.05 versus pooled MNC (pairwise comparisons).

*P <0.05 versus placenta (pairwise comparisons).

**Figure 1:**
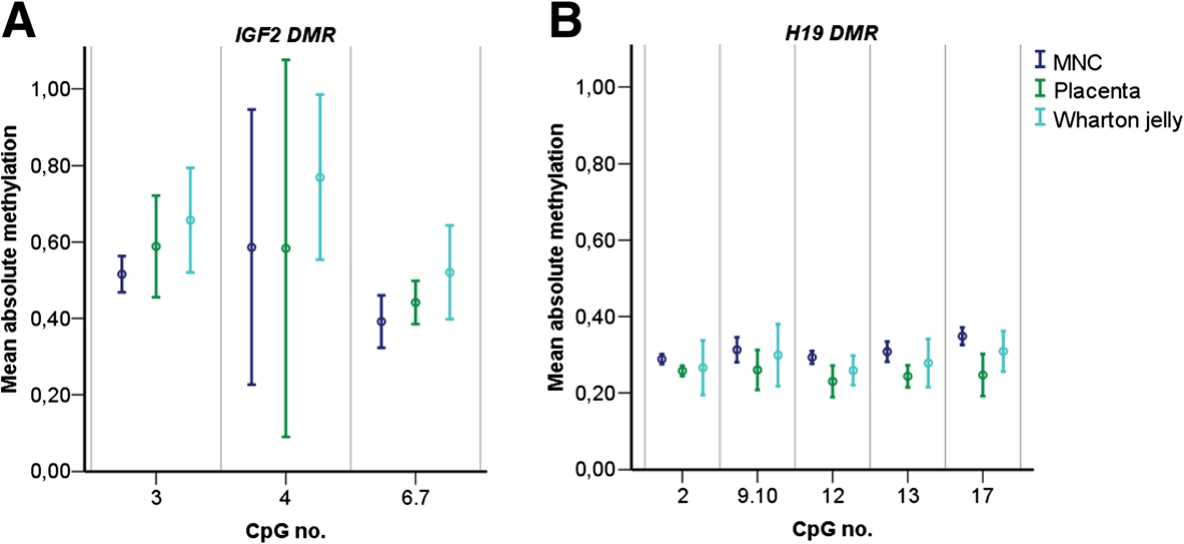
**Mean absolute DNA methylation levels per CpG site for the *IGF2 DMR *and *H19 DMR*.** Error plots of mean methylation levels (coloured dots) of all individuals ±2 standard deviations (coloured bars) shown for each CpG unit for each of the three tissues separately for (**A**) *IGF2 DMR* and (**B**) *H19 DMR*. *DMR*, differentially methylated region; MNC, mononuclear cells.

The mean *H19 DMR* methylation of both the placenta (*P* <0.001) and Wharton jelly (*P* <0.001) was statistically significantly lower than of MNC. This was similar in one out of five individual *H19 DMR* CpG units and only applied to placenta in two other CpG units. Wharton jelly was statistically significantly higher methylated than placenta in the *IGF2 DMR* (*P* = 0.032) and *H19 DMR* (*P* <0.001), as well as one individual *H19* CpG unit (Table 2 and Figure 1).

## Conclusion

This study provides a basic understanding of tissue-specific variation in DNA methylation of two imprinted genes in easily accessible tissues. The total MNC fraction of CD34^+^ and CD34^–^ appears rather homogeneous for DNA methylation analysis of these genes in umbilical cord blood. The observed between-tissue methylation differences seem to be small and could be explained either by consistently higher and lower methylation or by differences in sensitivity of tissues to environmental exposures, foetal and maternal factors. This needs further investigation in a larger sample size and therefore only careful conclusions should be drawn from these data.

Umbilical cord blood MNC are thus useful and easily accessible to study associations between epigenetic programming and pregnancy course and outcome, but do not always exactly reflect the methylation levels of other organs.

## Abbreviations

DMR: Differentially methylated region; H19: Imprinted maternally expressed transcript (non-protein coding); IGF2: Insulin-like growth factor 2; MNC: Mononuclear cells; PCR: Polymerase chain reaction; SNP: Single nucleotide polymorphism.

## Competing interests

The authors declare that they have no competing interests.

## Authors’ contributions

EH participated in the design of the study, collection of data, laboratory preparations and measurements, statistical analysis of data, and wrote the first version of the manuscript. JG contributed to the collection of data, laboratory work and writing of the manuscript. AR participated in the logistics of the laboratory preparations and contributed to the writing of the manuscript. LS supervised the designing of amplicons, laboratory logistics of the quality assessments of the DNA methylation measurements, and contributed to the writing of the final version of the manuscript. MV was involved in all laboratory work of the measurements of DNA methylation, and contributed to the final version of the manuscript. PE supervised the statistical analysis and contributed to the final version of the manuscript. JC was responsible for the laboratory logistics of the standardised collection and separation of MNC and contributed to the final version of the manuscript. ES was responsible for the clinical data collection and contributed to the final version of the manuscript. RS-T was responsible for all aspects of the study, participated in the design of the experiment and contributed to all versions of the manuscript. All authors approved the final version of the manuscript.
